# A decade of clinical microbiology: top 10 advances in 10 years: what every infection preventionist and antimicrobial steward should know

**DOI:** 10.1017/ash.2024.10

**Published:** 2024-01-25

**Authors:** Tulip A. Jhaveri, Zoe Freeman Weiss, Marisa L. Winkler, Alexander D. Pyden, Sankha S. Basu, Nicole D. Pecora

**Affiliations:** 1 Division of Infectious Diseases, University of Mississippi Medical Center, Jackson, MS, USA; 2 Division of Pathology and Laboratory Medicine, Tufts Medical Center, Boston, MA, USA; 3 Division of Geographic Medicine & Infectious Disease, Tufts Medical Center, Boston, MA, USA; 4 Division of Infectious Diseases, Emory University School of Medicine, Atlanta, GA, USA; 5 Division of Pathology and Laboratory Medicine, Lahey Hospital and Medical Center, Burlington, MA, USA; 6 Department of Anatomic and Clinical Pathology, Tufts University School of Medicine, Boston, MA, USA; 7 Department of Pathology, Brigham and Women’s Hospital, Boston, MA, USA

## Abstract

The past 10 years have brought paradigm-shifting changes to clinical microbiology. This paper explores the top 10 transformative innovations across the diagnostic spectrum, including not only state of the art technologies but also preanalytic and post-analytic advances. Clinical decision support tools have reshaped testing practices, curbing unnecessary tests. Innovations like broad-range polymerase chain reaction and metagenomic sequencing, whole genome sequencing, multiplex molecular panels, rapid phenotypic susceptibility testing, and matrix-assisted laser desorption ionization time-of-flight mass spectrometry have all expanded our diagnostic armamentarium. Rapid home-based testing has made diagnostic testing more accessible than ever. Enhancements to clinician-laboratory interfaces allow for automated stewardship interventions and education. Laboratory restructuring and consolidation efforts are reshaping the field of microbiology, presenting both opportunities and challenges for the future of clinical microbiology laboratories. Here, we review key innovations of the last decade.

## Introduction

**Figure 1.**
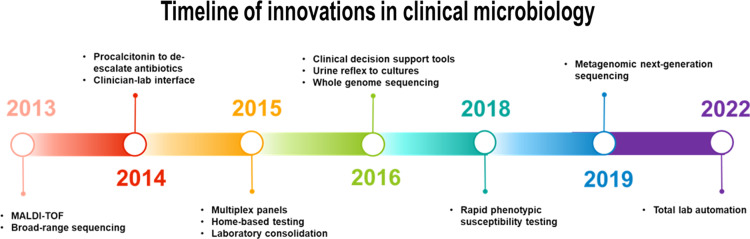
Timeline demonstrating the top innovations over the last 10 years. Though some technologies were developed prior to 2013, these dates reflect their emergence in mainstream clinical microbiology.

The past 10 years have brought paradigm-shifting changes to clinical microbiology. This paper explores the top 10 transformative innovations across the diagnostic spectrum, including not only state of the art technologies but also preanalytic and post-analytic advances (Table 1). Clinical decision support tools (CDST) have reshaped testing practices, curbing unnecessary tests. Innovations like broad-range polymerase chain reaction (PCR) and metagenomic sequencing, whole genome sequencing (WGS), multiplex molecular panels, rapid phenotypic susceptibility testing, and matrix-assisted laser desorption ionization time-of-flight mass spectrometry (MALDI-TOF MS) have all expanded our diagnostic armamentarium. Rapid home-based testing has made diagnostic testing more accessible than ever. Enhancements to clinician–laboratory interfaces allow for automated stewardship interventions and education. Laboratory restructuring and consolidation efforts are reshaping the field of microbiology, presenting both opportunities and challenges for the future of clinical microbiology laboratories. Herein, we categorize these laboratory advances as preanalytic, analytic, post-analytic, and other to reflect how these would be implemented in clinical care. A timeline is provided to demonstrate when in the past 10 years these technologies or innovations emerged (Fig. 1).

**Table 1.** Overview of the top ten innovations in clinical microbiology over the past decade, highlighting their applications, key benefits, and associated challenges

	Innovation area	Applications	Key benefits	Challenges/Limitations
Preanalytic	Clinical decision support tools	Best-practice alerts Guidelines and algorithms Indication selection using guided test ordering Change in order sets	Drive appropriate test selection Prevent overutilization of tests in low-impact situations	Decreased testing when actually indicated Alert fatigue leading to clinicians overriding alerts Provider and IT pushback
	Host Pathogen Response	Inflammatory markers (Procalcitonin) Urinalysis Reflex to Culture	Antibiotic discontinuation Diagnostic stewardship	Specificity and Reproducibility Utilization management Integration with microbiology
Analytic	Sequencing	Broad range PCR, targeted NGS Metagenomic sequencing Whole genome sequencing	Identification of organisms directly from clinical specimens, even when culture negative High species level resolution for organism identification Determine strain relatedness for epidemiological purposes	Sensitivity and specificity dependent on preanalytic factors Results can be difficult to interpret when commensal organisms or contaminants are identified Unknown how to report and act upon WGS data in real-time Does not provide phenotypic susceptibility data
	Multiplex panels	Syndromic-based testing	Antibiotic stewardship Avoid decision fatigue	Positive results not always clinically relevant Expense
	Rapid susceptibility testing	Novel methods of rapid susceptibility testing	Guides early selection and use of optimal antibiotics	Requires adjudication of discrepancies between rapid AST and finalized traditional AST results
	MALDI TOF MS	Bacterial, fungal identification from isolates	Improved accuracy Shorter turnaround time	Capital costs Over-reporting
	Home testing	Rapid home-based antigen tests	Convenience Privacy Access	Test performance and result interpretation Potential cost per test Quality control of the testing components Linkage to care and inclusion in the EMR Tracking of any results that are of concern for public health
Post analytic	Clinician-lab interface	Framing Cascade reporting Selective reporting Result review and feedback	Guides appropriate decision-making following test results Automates stewardship interventions and education	Limiting clinician’s input leading to missed diagnosis
Other	Laboratory consolidation	Acquisition by commercial laboratories Centralized/localized testing within a health system Total laboratory automation	Increased cost savings and efficiency Concentration of resources/expertise/ technology within a network to provide access to highest quality across the system Uniform adherence to stewardship best practices and guidelines	Increased turnaround time for results to remote sites Logistical challenges, such as specimen stability Risk for financial considerations to drive decisions at the expense of patient safety or quality

## Preanalytic

### Clinical decision support tools

Clinical microbiologists have known for a long time that preanalytical issues are often the most important, yet overlooked, factors in producing high-quality results. For instance, emphasizing good specimen collection using appropriate techniques and having quality criteria for working up cultures from non-sterile sites. The focus of preanalytic innovations over the past decade has shifted to behavioral economics using automated system-based CDST to nudge clinicians earlier in clinical workup for better utilization of diagnostic tests.^1,2^ Antimicrobial stewardship programs combined with information technology staff have played a crucial role in the design and implementation of CDST. Some examples of these novel interventions include:

- Best-practice alerts (BPAs) to stop unnecessary diagnostic testing in patients without symptoms of infection. For example, firing BPAs when ordering urine testing in asymptomatic patients led to reduced urine culture ordering and reduced antibiotic orders^3^ Utilizing Electronic Medical Record (EMR) hard and soft stops if a patient is not meeting criteria for *Clostridium difficile* testing: <1 year old, laxatives within 48 hours, or < 3 loose stools in 24 hours led to reduced testing and positive clinician perception.^4,5^.
- Guidelines and algorithms to promote appropriate testing practices. Examples include algorithms to reduce unnecessary blood culture collection^6,7^ or urine culture reflex based on predefined urinalysis criteria to reduce unnecessary workup for urine cultures and administration of antibiotics.^8^
- Indication selection using restrictive or guided test ordering via built-in EMR algorithms to drive appropriate test selection. Examples include requiring a clinician to input an approved indication on an order set before ordering a urine culture,^9,10^ endotracheal aspirate culture,^11^ rapid multiplex respiratory pathogen panel,^12^ or rapid multiplex meningitis panel.^13^
- Change in order sets—for instance, removing urine cultures from standard admission order sets to prevent overutilization in low-yield situations led to decreased urine cultures ordered^14,15^

Challenges: These interventions may inadvertently reduce testing in situations where it is indicated, for instance, not obtaining urine cultures in asymptomatic pregnant women. Most importantly, implementation challenges such as “alert fatigue” due to overuse of electronic reminders disrupting usual workflow or “provider pushback” due to conflicts between CDST recommendations and provider expertise or beliefs lead to clinicians ignoring or overriding these alerts.^16,17^ Resources such as information technology staff are additionally utilized during integration of these tools into clinical practice.

### Host–pathogen response

Integration of the host immune response to pathogens into infectious disease diagnostics has seen substantial expansion in the past decade. Two of the major areas of growth include:

- Monitoring of inflammatory markers—the most notable change is the use of procalcitonin for guidance in discontinuation of antibiotic treatment in certain patient populations (critically ill, lower respiratory tract infections, and in certain pediatric populations).^18^
- Incorporation of cell counts and differentials in guiding specimen adequacy and likelihood of infection—this has been most notably used in urinalysis reflex to urine culture, in which demonstration of pyuria (commonly defined in urine as >10 WBC/hpf) is required before performing urine culture. This has led to a significant decrease in unnecessary workup of urine isolates.^19,20^ Similar approaches to using cell counts or inflammatory markers have been utilized in the workup of meningitis/encephalitis as well as periprosthetic joint infection. Although the methodology for measurement has not changed dramatically in the past decade, their utilization in stewardship and influence on the workup of microbiology specimens has considerably increased in the past 10 years and is now the standard of care.

Challenges: Specificity remains a significant limitation to using host–pathogen response markers to guide treatment. Treatment markers such as C-reactive protein (CRP) and procalcitonin can be elevated in a variety of infectious and inflammatory conditions, and despite their long standing use, are often improperly used.^21,22^ Inclusion of additional markers such as tumor necrosis factor-related apoptosis-inducing ligand (TRAIL) and other proteins have helped refine this approach but is still early in its use.^23^ Another challenge is a lack of integration between the microbiology laboratory and the laboratories that monitor the host immune response (eg, CRP, procalcitonin in chemistry, cell counts in hematology). Closer interactions between clinical laboratory sections will be crucial in the successful utilization of these approaches.

## Analytic

### Broad-range sequencing

Various sequencing methods have become integral for identifying organisms and defining taxonomy, whether directly from specimens or from cultured isolates.^24^ In microbiology labs, initial efforts are made to identify isolates using conventional techniques. However, if these conventional methods prove unsuccessful, labs have the option to either perform sequencing in house (if they have the capability) or send isolates to reference labs for a definitive identification through sequencing. Sequencing results are often used as the “gold standard” for species-level identification.

Despite being colloquially referred to as “universal PCR,” broad-range PCR is restricted to selecting ribosomal subunits specific to either fungi or bacteria. Additional broad-range targets, such as *rpoB*, may be used to further differentiate among groups such as acid-fast bacteria.^25^ Some laboratories will require clinicians to choose the relevant targets for testing. Fresh frozen tissue is generally the preferred specimen. It is also possible to perform sequencing on formalin-fixed paraffin-embedded tissue (FFPE). The sensitivity of sequencing from FFPE specimens may be compromised or reduced after the fixation process. In general, samples with concurrent pathology that do not demonstrate histopathological evidence of infection are unlikely to yield positive results by sequencing and efforts should be made to discourage sequencing in these scenarios.^26,27^

Challenges: Sequencing is not available in most clinical microbiology laboratories. Commercially available options, such as University of Washington or Mayo Clinical Laboratories, are often expensive and the overall turnaround time can range from 1 to 4 weeks. Because most laboratories only send sequencing in instances where traditional microbiological methods are negative or inconclusive, clinical laboratories must have some way of retaining, tracking, and freezing specimens where sequencing is requested, but initial results are still pending (to avoid accidentally discarding specimens). There is variability in sensitivity as compared to culture-based methods, depending on the specimen source, fixation process, and correlative histopathology. Sequencing results must be correlated clinically, usually by a clinical microbiology or infectious disease clinician, as sequencing of contaminants may occur and result in provider confusion.

### Metagenomic Next-Generation Sequencing (mNGS) and Whole Genome Sequencing (WGS)

In recent years, the use of mNGS (a massively paralleled, rapid, high throughput method of sequencing all the genetic material in a clinical sample) and WGS (sequencing of entire organism genomes) has entered into mainstream clinical use.^28,29^ These technologies have generally required significant bioinformatic/computational analysis and, outside of large academic medical centers, are typically confined to reference laboratories. This is starting to change with commercially available software solutions.

mNGS assays can be used directly on primary specimens. For example, NGS metagenomics can be performed on CSF and may be used for detection of RNA viruses.^30^ In recent years, there has been increased interest in organism identification directly from blood samples, bypassing culture incubation steps. NGS directly from plasma is available commercially, using cell-free DNA sequencing (sequencing small fragments of DNA released into the bloodstream) to identify pathogens both within the bloodstream and at distant sites of infection. This novel concept of a “liquid biopsy” is gaining popularity, especially in identifying organisms when traditional methods are negative or where invasive biopsy is contraindicated.^31^

Pathogen WGS (sequencing from pure or highly enriched isolate preparations) is also entering mainstream clinical microbiology laboratories. WGS is most routinely used to characterize bacterial specimens for purposes such as high-resolution identification of unusual isolates, investigations into novel antimicrobial resistance genes, and assessments of relatedness for infection control and epidemiological purposes.^32^ Although fungal WGS is still a developing method, viral WGS became a critical tool to understand epidemiology and spread during the COVID-19 pandemic.^33^

Challenges: Sequencing of clinical specimens presents a number of challenges that limit widespread use of these technologies. Besides high costs and variable turnaround times,^34^ the lack of understanding of what pathogens are being tested and the optimal timing of testing may cause clinicians to not order appropriate testing (eg, when suspecting certain viruses or parasites that are not included in the reference NGS database) or miss the window of opportunity for optimal testing (eg, most arboviral infections do not exhibit detectable levels of RNA in the CSF beyond the first 1-2 weeks after the infection’s onset, limiting the sensitivity of this test in the later diagnostic stages^35,36^). Clinicians may erroneously want to use mNGS sequencing methods to “rule out infection”^37^ despite lack of data on use for this indication. Most importantly, test interpretation and reporting remain a critical problem. mNGS detection of multiple organisms within a clinical sample may often include detection of commensal or nonpathogenic organisms that may be misleading to clinicians and lead to excessive treatment^38^ or additional diagnostics that would not have otherwise been ordered.^37,39,40^ Adjudication by clinical microbiologists or infectious disease physicians should be performed in all cases.

WGS from cultured isolates also produces an incredibly rich data set; however, the real-time clinical utility of WGS data is still an area in need of development. Challenges include predicting the phenotype of genomic antibiotic resistance results and understanding the threshold for relatedness when comparing genomes from potentially related strains as part of outbreak investigations. There are lack of clear guidelines as to which genetic information should be reported to the clinician and little guidance on how this information should be acted on, if at all.

### Multiplex panels

Multiplex PCR panels are commercially available for multiple specimen sources including upper respiratory tract, lower respiratory tract, blood, stool, prosthetic joint, abdominal, and genitourinary tract (Table 2). These panels include multiple organism targets common to a particular infectious syndrome. This can reduce cognitive error when providers are required to order multiple tests separately and their rapid turnaround time can theoretically reduce broad-spectrum antimicrobial use, though this has not consistently been found to be the case in studies unless testing is coupled with antimicrobial stewardship feedback.^41^ The maximal benefit of these tests is realized if they are able to be run on all shifts, with relatively short turnaround times, which can cause significant logistical issues for laboratories, particularly given current staffing shortages.^42,43^

**Table 2.** Summary of FDA approved commercially available multiplex assays including targets, source, methodology, turnaround time, and clinical caveats

Blood								
Name	Manufacturer	Organism targets	Gene targets resistance genes (RGs)	Specimen source	Technique	Turnaround time	Clinical caveat	Citation
Biofire Blood culture identification (BCID) 2 panel	Biomerieux	7 yeast, 26 bacterial	10 RGs	Positive blood culture bottle	mPCR	1 hour	Detects multiple targets in polymicrobial samples	^95^
ePlex BCID Gram-positive panel	GenMarkDx	20 organisms, pan-Gram-negative and pan- Candida	4 RGs	Positive blood culture bottle	mPCR	1.5 hours	Detects multiple targets in polymicrobial samples	^96^
ePlex BCID Gram-negative panel	GenMarkDx	21 organisms, pan-Gram- positive and pan-Candida	7 RGs	Positive blood culture bottle	mPCR	1.5 hours	Detects multiple targets in polymicrobial samples	^96^
ePlex BCID Fungal panel	GenMarkDx	15 organisms	None	Positive blood culture bottle	mPCR	1.5 hours	Detects multiple targets in polymicrobial samples	^96^
Verigene Gram-positive panel	Diasorin	13 organisms	3 RGs	Positive blood culture bottle	Nanoparticle probe	2.5 hours	Unreliable for polymicrobial samples	^97^
Verigene Gram-negative	Diasorin	9 organisms	6 RGs	Positive blood culture bottle	Nanoparticle probe	2.5 hours	Unreliable for polymicrobial samples	^97^
T2Bacteria panel	T2Biosystems	5 organisms	None	Direct from blood	NMR	3-5 hours	Lower limit of detection	^98^
T2Candida panel	T2Biosystems	5 organisms	None	Direct from blood	NMR	3-5 hours	Lower limit of detection	^99^
Accelerate Pheno and blood culture panel	Accelerate diagnostics	14 bacteria, 2 yeast	Susceptibility to 17 agents	Positive blood culture bottle	PNA-FISH, morphokinetic cellular analysis	2-7 hours	Unreliable if multiple organism morphologies or polymicrobial samples	^100^
**Upper respiratory**								
Name	Manu-facturer	Organism Targets	Gene targets	Specimen source		Turnaround time	Cost	
Biofire FilmArray respiratory pathogen panel	Biomerieux	17 viruses, 3 bacteria	None	Nasopharyngeal (NP) swab	mPCR	1 hour	Requires pairing with ASP* to reduce antimicrobial utilization	^101^
Biofire Respiratory 2.1-EZ panel	Biomerieux	15 viruses, 4 bacteria	None	NP swab	mPCR	45 minutes	Requires pairing with ASP to reduce antimicrobial utilization	^102^
Verigene Respiratory pathogens flex	Luminex	13 viruses and 3 bacteria	None	NP swab	mPCR	2 hours	Requires pairing with ASP to reduce antimicrobial utilization	^103^
eSensor Respiratory virus panel	GenMarkDx	14 viruses	None	NP swab	mPCR	6 hours		^104^
ePlex Respiratory virus panel 2	GenMarkDx	16 viruses, 2 bacteria	None	NP swab	mPCR	3 hours	Requires pairing with ASP to reduce antimicrobial utilization	^104^
**Lower respiratory**								
Biofire FilmArray pneumonia panel	Biomerieux	8 viruses, 18 bacteria	RGs	Sputum or BAL	mPCR	1 hour	Requires pairing with ASP to reduce antimicrobial utilization	^105^
**Gastrointestinal**								
Biofire FilmArray GI panel	Biomerieux	5 viruses, 11 bacteria, 4 parasites	None	Stool	mPCR	1 hour	Cannot differentiate between live and dead organisms, high rates of colonization with unclear clinical significance	^106^
xTAG GI Pathogen panel	Luminex	3 viruses, 8 bacteria, 1 bacterial toxin, 3 parasites	None	Stool	mPCR	5 hours	Cannot differentiate between live and dead organisms, high rates of colonization with unclear clinical significance	^107^
BDMax Enteric bacterial panel	BD	4 bacteria	None	Stool	mPCR	3.5 hours	Cannot differentiate between live and dead organisms	^108^
BDMax Extended enteric bacterial panel	BD	8 bacteria	None	Stool	mPCR	3.5 hours	Cannot differentiate between live and dead organisms	^109^
BDMax Enteric viral panel	BD	5 viruses	None	Stool	mPCR	3 hours	Cannot differentiate between live and dead organisms	^110^
BDMax Enteric parasite panel	BD	3 parasites	None	Stool	mPCR	4.5 hours	Lower sensitivity than conventional methods	^111^
Verigene Enteric pathogens panel	Diasorin	2 viruses, 5 bacteria, 2 bacterial toxins	None	Stool	mPCR	2 hours	Cannot differentiate between live and dead organisms, high rates of colonization with unclear clinical significance	^112^
**Joint**								
Biofire joint infection panel	Biomerieux	15 Gram-positive, 14 Gram-negative, 2 yeast	20 RGs	Synovial fluid	mPCR	1 hour	Missing many common causes of prosthetic joint infections	^113^
**CNS**								
Biofire meningitis/Encephalitis panel	Biomerieux	7 viruses, 6 bacteria, 1 yeast	None	Cerebrospinal fluid	mPCR	1 hour	Cannot fully replace traditional diagnostics	^114^

Note.

* ASP: antibiotic stewardship program.

Challenges: There are downsides to the use of multiplex panels. One of these is significant testing costs, which are inconsistently covered by insurance, leading to either a high bill to the patient or a high cost to the laboratory. Additionally, all components of these panels are not necessarily understood by all clinicians, which can create confusion and inappropriate additional testing or treatments without clear interpretation guidelines. Panels are often not customizable, though some companies offer panels with extension options.^44^ Excessive targets may result in multiple positive targets that do not always fit the clinical picture, such as with stool multiplex panels^45^ and the detection of colonizing forms of *Clostridium difficile* ^46^ or species of *Escherichia coli* that lack clinical guidelines regarding treatment or significance (Enteroaggregative *E. coli*, Enteropathogenic *E. coli*). Positive results may lead to confusion from patients and providers or lead to unnecessary treatment. These testing panels have significant promise to decrease testing turnaround time and improve diagnostic accuracy, but their successful implementation requires stewardship integration for test appropriateness and interpretation.

### Rapid phenotypic susceptibility

In addition to rapid detection of molecular targets, another advance has been the introduction of rapid phenotypic susceptibility testing. Traditional antimicrobial susceptibility testing (AST) can take 24–48 hours to result after isolation of cultured organisms. To expedite this process, rapid AST can be performed manually by direct use of positive blood culture broth as the inoculum for conventional methods using disk diffusion techniques with results in up to 6 hours.^47^ However, this is a laborious practice and not widely used. Novel, less labor-intensive phenotypic susceptibility methods that mimic results produced by standard AST have become commercially available and represent an area of rapid growth. Novel methods include detection of changes in cell morphology induced by antimicrobials using single cell microscopy, assessing the rate of cell division, examining gene expression patterns, or detection of volatile organic compounds.^48^ One commercially available system in the US performs AST approximately 7 hours from positive blood cultures using time-lapse imaging under dark-field microscopy, monitoring morphological and kinetic changes in the bacteria to determine MICs.^49^ Several other rapid AST platforms are in the development pipeline. Rapid AST should be paired with rapid identification, as MIC results and their correlative breakpoints are only interpretable when paired with the organism ID. Institutions may choose platforms that pair rapid ID with rapid AST or use separate instruments to achieve this.

Challenges: Implementation of rapid AST methods requires monitoring and adjudication of discrepancies between rapid AST and finalized traditional AST results if both methods are performed.^50^ Institutions should consider how they will alert providers or stewardship teams of rapid results, particularly if rapid AST is run on all shifts in institutions that are used to receiving updated susceptibility reports only during day shift hours.

### MALDI-TOF MS

Perhaps the most impactful technological innovation in the clinical microbiology laboratory over the past decade has been the introduction of matrix-assisted laser desorption/ionization-time-of-flight mass spectrometry (MALDI-TOF) for the identification of routine clinical bacterial and fungal isolates. This technique involves the untargeted proteomic spectral analysis directly from bacterial or fungal colonies and rapid species-level identification through a growing highly diverse database. The first FDA-approved MALDI-TOF MS instruments were introduced in 2013, and since that time, these platforms have largely replaced many of the time and labor-intensive biochemical methods that had been the primary method of identification in laboratories for over a half century. In addition to more accurate species identification, MALDI-TOF MS provides significant reduction (12–48 hours) in turnaround times for identification.^51,52^ More recent and ongoing advances involve the application of the methodology for acid-fast bacteria, Nocardia,^53^ and mold identifications^54^ as well as applications in epidemiological investigations and antibiotic resistance.^55^

Challenges: Despite this significant impact and low reagent costs, the relatively high capital costs of MALDI-TOF MS instrumentation have slowed its adoption, particularly in smaller laboratories. Also, the discontinuation of conventional biochemical assays results in a lack of robustness during planned or unplanned instrument “downtimes.” Finally, its ease of use coupled with its rapid and accurate identification to species level has resulted in reporting of organisms, which may not have been easily reported in the past, such as certain coagulase-negative staphylococcal or alpha-hemolytic streptococcal species. This “overreporting” can lead to confusion amongst clinicians not familiar with these organisms and may lead to an increase in their unnecessary treatment. Therefore, the inclusion of laboratory and antimicrobial stewardship is recommended during implementation.

### Home-based testing

The COVID-19 pandemic accelerated what was already a widespread interest in patient-centered infectious disease testing, including samples which are patient collected but analyzed by a clinical laboratory as well as fully home-based testing.

Patient-collected samples have become a popular method for diagnosing sexually transmitted infections, including *Neisseria gonorrhoeae*, *Chlamydia trachomatis*, and *Trichomonas vaginalis*.^56–58^ With the COVID-19 pandemic, self-collection of anterior nares (AN) specimens for respiratory viral testing^59^ was also utilized in many situations, though concerns for the sensitivity of AN swabs for viruses such as RSV^60^ and adenovirus^61^ may limit respiratory self-testing for other infections. Convenience, privacy, and access are major advantages of self-collected specimens, and analyses of self-collection tend to support both the quality of the result as well as a positive effect on test uptake/utilization.^62^ Linking self-collected specimens with telemedicine (rather than traditional clinic visits with a provider) enhances access as well as speed and flexibility.^63,64^ In addition to established healthcare routes that incorporate self-sampling with and without telemedicine, there are also several companies that provide self-collection kits for sexually transmitted diseases, respiratory viruses, and urinary tract infections, distributed in the context of remote providers.^65^

Challenges: The discussion around home-based testing without associated telemedicine visits is far more complex. Fully home-based FDA-cleared infectious disease testing had been confined to assays for HIV before at-home COVID and COVID/Influenza tests became available under Emergency Use Authorization (EUA). To allow for kit stability, ease of use, and clear interpretation, home-based tests are usually antigen-detection lateral-flow assays that wick a patient’s sample across test and control zones and deliver a result through the appearance of a colored spot or line. Generally speaking, these tests do not have the same sensitivity (and often specificity) as molecular assays^66^ and a great deal of debate has occurred over what constitutes sufficient sensitivity.^67^ During the COVID-19 pandemic, at-home molecular assays were also developed and are a promising tool for future at-home testing.^68^ Concerns about at-home testing include test performance and result interpretation, potential cost per test (often out of pocket),^69^ quality control of the testing components/process, linkage to care and inclusion in the EMR, and tracking of any results that are of concern for public health.^70,71^

The public focus on at-home testing and telemedicine during the COVID-19 pandemic has brought the issues surrounding both self-collected laboratory testing and at-home testing into the spotlight. The challenges of self-collected testing largely involve sample/transport device stability/performance and quality of sample collection.^65^ The advantages of privacy, agency, convenience, and increased test uptake are so substantial that, despite concerns about quality and care linkage, it is expected that these patient-centered testing approaches will expand in the future.^72^

## Post-analytic

### Clinician/laboratory interface

Another significant advancement in the clinical microbiology laboratory has been the enhancement of the clinician–laboratory interface with the help of nudging strategies to guide appropriate decision making while maintaining prescriber autonomy.^73^ Some examples of these novel interventions include:

- Framing: combines results with free text or educational materials to provide context for the results, changing their relative attractiveness. Examples include adding interpretative guidance on respiratory cultures growing normal commensal flora with a comment “no Methicillin-resistant *Staphylococcus aureus* or *Pseudomonas aeruginosa* isolated^74^,” adding a nudge on a positive *C. difficile* nucleic acid amplification test with negative toxin enzyme immunoassay test to “consider colonization or early infection,”^75^ adding interpretation guidance for coagulase-negative staphylococci growing in one of four blood culture bottles (one of two sets) as “possible contaminant,” and adding an interpretive comment on respiratory cultures for β-lactamase-negative *Haemophilus influenzae* or *Moraxella catarrhalis* stating, “this organism is predictably susceptible to ampicillin or amoxicillin.”^76^
- Cascade reporting: reports narrow-spectrum agents initially with subsequent susceptibilities reported only on resistant organisms, for example, reporting only ceftriaxone on ceftriaxone-susceptible *Escherichia coli* and *Klebsiella* species^77^ and only cefazolin on cefazolin-susceptible gram-negative organisms.^78^ The goal of these interventions is often to reduce the use of broad-spectrum agents like meropenem or antibiotics with high risk for *C. difficile* infection or other antibiotic-associated adverse events, like fluoroquinolones.^79^
- Selective reporting: restricts reporting of susceptibility results of certain antimicrobials based on predefined criteria (ie, broad-spectrum antimicrobials and high adverse drug events). Examples include suppression of ciprofloxacin susceptibility for Enterobacterales, for all sites of infection, when there was susceptibility to other agents on the gram-negative susceptibility testing panel,^80^ or in its most extreme form, not reporting urine culture results from noncatheterized inpatients, instead requiring clinicians to call the clinical microbiology lab for results if concerns for true infection persist.^81,82^
- Result Review and Feedback: results of blood culture rapid diagnostics are reported with real-time decision support using antimicrobial stewardship personnel to assist in interpretation at the time of medical decision making. Studies have been published using this with staphylococcal blood-stream infections (BSI),^83^ gram-negative BSI,^84,85^ and all BSI.^86^ These interventions are known to improve patient outcomes^87^ and are cost effective.^88^

Challenges: although the aim of these nudging strategies is to improve diagnostic processes to prompt timely action, there is limited evidence to show that they decrease antimicrobial use. More prescriptive interventions could limit clinician input and lead to missed diagnoses.

## Other

### Laboratory consolidation

Health system consolidation is a growing trend that has impacted clinical laboratories in recent years encompassing all phases of testing. Laboratory consolidation came into prominence with the development of large commercial laboratories.^89^ As hospital laboratories have shifted from revenue to cost centers, these commercial laboratories have increasingly sought to purchase hospital laboratories. Some laboratory medicine departments have alternatively navigated a solution to consolidate various laboratory tests and functions within their growing health systems.^90^ This solution often entails one central flagship laboratory taking on a larger volume of testing, allowing the health system to concentrate resources toward recent technologies and advancements mentioned earlier in this review in one location, which may allow a cost-efficient mechanism for improvements in quality to reach smaller hospitals in the network.

Thus, consolidation both depends on and facilitates related advancements, a prime example of which is total laboratory automation (TLA). TLA has been particularly impactful for microbiology laboratories, which traditionally have maintained highly complex, manual, and time-intensive test menus. Thus, TLA may allow health systems to continue offering this testing to increasing volumes of patients around the clock as the workforce of qualified technologists continues to shrink, although robust comparisons of outcomes in automated laboratories remain lacking in the literature. Consolidation provides the opportunity for coordination and standardization of such activities, as well as optimal adherence to best practices, reporting, and turnaround time across a network^91^; TLA likewise may facilitate access to results via technologies such as remote visualization of culture plates.^89^

Challenges: A major drawback of laboratory consolidation is the loss of access of providers to the laboratory. This results in disengagement between the two groups and impedes education, consultation, and the close engagement required for policy development in antimicrobial stewardship and infection prevention. Off-site centralization may lead to delays or quality of care. For example, blood culture incubation and workup are a highly complex and resource-intensive process that is increasingly targeted for centralization. Though guidelines suggest that specimens must be placed into incubation systems within two hours of collection to prevent false-negative results,^92^ significant delays are common when samples are transported to centralized locations. Although technology is increasingly available to mitigate the impact of delayed incubation,^93^ turnaround time remains of paramount importance for blood cultures. Throughout the process of laboratory consolidation, medical directors and administration leadership must work together to maintain an appropriate balance between financial considerations and patient safety.

## Conclusion

We have summarized top advances made in the field of clinical microbiology in the past decade (Table 1) that every antimicrobial steward and infection prevention practitioner should know. To justify the costs of incorporating these novel microbiology advances into clinical practice, it is essential for clinicians to utilize these techniques appropriately. For example, developing institution-specific guidelines would be one way to support these key diagnostic and antimicrobial stewardship initiatives.^94^ This would require a highly collaborative and interdisciplinary approach by working synergistically with key stakeholders including clinical microbiologists, infectious disease specialists, antimicrobial stewards, infection preventionists, hospitalists, primary care physicians, and healthcare information technology teams.
